# Effect of informal care on health care utilisation for the elderly in urban and rural China: evidence from China health and retirement longitudinal study (CHARLS)

**DOI:** 10.1186/s12913-022-07675-2

**Published:** 2022-03-01

**Authors:** Xinlan Chen, Dai Su, Xinlin Chen, Yingchun Chen

**Affiliations:** 1 Department of Health Management, School of Medicine and Health Management, Tongji Medical College, Huazhong University of Science and Technology, Wuhan, 430030 China; 2 Key Research Institute of Humanities & Social Sciences of Hubei Provincial Department of Education, Research Centre for Rural Health Service, Wuhan, 430030 China; 3 Department of Health Management and Policy, School of Public Health, Capital Medical University, Beijing, China

**Keywords:** Informal care, Health care utilization, Rural China

## Abstract

**Background:**

Receiving informal care from family members is the mainstream way of care for the elderly in China because of the influence of the culture of filial piety. However, the relationship between informal care and health care use in urban and rural areas needs to be further explored. This study aimed to understand the association between informal care and health care utilisation for the elderly and explore how this effect may differ between urban and rural China.

**Method:**

A total of 5704 residents aged 65 years and above were selected from wave 3 (2015) and wave 4 (2018) of the China Health and Retirement Longitudinal Study, which is a nationally representative survey. A negative binomial regression model for the panel data was used to explore the relationship between informal care and health care utilisation. A fixed-effect binary choice model for panel data was used for the sensitivity test.

**Result:**

The elderly who received informal care had increased in outpatient and inpatient visits compared with those who did not receive informal care. The inpatient visits of the elderly who received 15–29 days of informal care was higher than the elderly who did not receive informal care (incidence rate ratio [IRR] = 2.082, *P* < 0.05). Moreover, the elderly who received informal care for more than 30 days had 39.6% more inpatient visits (IRR = 1.396, *P* < 0.01) and 37.4% more outpatient visits than the elderly who did not receive informal care (IRR = 1.374, *P* < 0.05). For urban respondents, receiving informal care can facilitate outpatient use of the elderly, but for rural respondents, receiving informal care can predict an increase in outpatient and inpatient visits.

**Conclusion:**

Informal care was associated with higher use of health services. The association between informal care and health care utilisation varies between rural and urban residents. These findings indicate the role of informal care and remind that relevant departments should pay attention to the differences in medical service utilisation levels amongst different elderly groups.

## Background

Along with global ageing, China has stepped into the ageing society since 2000, and China’s ageing process is much faster than those of other low- and middle-income countries. According to the results of the seventh census, 13.8% of the population is over 65 years old in 2020 [1]. However, over 17% of the elderly population should be in the hospital, but failed to be hospitalised [2]. Unlike developed countries, China, as a developing country, has not effectively fulfilled the medical service needs of the elderly (especially the elderly in rural areas) [3, 4]. Informal care provided by relatives, such as children and spouses, is currently the most mainstream way of care in China because of the culture of filial piety. Filial piety is a core value of Confucianism in Chinese culture and has a strong influence; thus, many Chinese elderly will not choose to move into nursing homes [5]. China’s recent documents clearly supported the continuous improvement of home-based, community-based, institutional-based and integrated elderly care service systems which promotes the health of the elderly and the development of the elderly care industry [6, 7]. However, informal care will still be an important part of China's long-term care system for some time.

Rural and urban China represented two distinctive classes. For instance, compared with their urban counterparts, rural residents are more likely to be farmers with lower levels of education and income [8]. Urban and rural areas may differ in family structure. The children of urban families visit their parents more conveniently and frequently, whereas the children of rural families only meet with their parents on festivals due to distance issues [9]. In China, just as there are many economic and social differences between urban and rural areas, there are also obvious differences in elderly care between urban and rural areas. Compared with the urban elderly, the rural elderly is more dependent on informal care from family members. In rural China, informal care plays an absolutely dominant role, while other care models are seriously underdeveloped. Moreover, the accessibility of health services for the elderly in rural areas is worse than for those in urban areas [10]. In this case, the scarcity of studies that investigated the patterns of informal care and formal care by considering rural–urban disparity warrants the identification of the relationship in the specific rural and urban contexts to improve the validity of the findings.

Previous studies on informal and formal care do not definitively establish whether informal care is positively or negatively associated with formal care. Some studies found through carefully testing and controlling for endogeneity that informal care by children can reduce the use of home health care and nursing home care, as well as hospital care and physician visits [11, 12]. Furthermore, the care provided by children can reduce the expenditure on health services for the elderly [13, 14]. Previous studies also show that informal care by adult children can increase high-skilled home care use (nursing/personal care) in Europe [15]. Accepting the care provided by family members can improve the use of outpatient and hospitalisation by the elderly, especially for those over 75 years old [16]. These studies do not reach uniform conclusions about the effect of informal care on health service utilisation by the elderly. Moreover, no study has considered the heterogeneity between urban and rural elderly. The dual structure of urban and rural areas in China leads to the difference in the care system between urban and rural areas. Therefore, further work is required, such as longitudinal studies in this area to gain a better understanding of the role of informal care [17]. Research on the relationship between family care and medical care in China can help to further understand the value and contribution of unpaid family care to the health care utilisation of the elderly in urban and rural areas, provide policy support for improving the function of family care and better cope with the challenges of ageing.

Our study expands on the literature by examining how informal care affects the frequency of outpatient and inpatient use of the elderly in urban and rural China by using negative binomial regression for panel data and considering the total days of informal care provided by all family members. This research answers the following questions: Is informal care positively or negatively associated with health care utilisation? Is there a difference in this association between China's urban and rural areas? The specific hypotheses tested were: (1) Informal care received by the elderly is positively associated with health care utilisation; (2) The association between informal care and health care utilisation differs between urban and rural areas. Informal care makes use of formal care more likely, especially in rural areas.

### Conceptual model

This study is based on the Andersen and Newman Framework of Health Service Utilisation. The Andersen model is used in analysing the factors which may affect health service utilisation (including hospitalisation, doctor visits, dental care and so on). The initial Andersen model includes predisposing characteristics, enabling resources and needs [18]. Amongst them, predisposing characteristics refer to the demographic characteristics, social structure and health beliefs of the population who tend to use medical services before the occurrence of a disease. Enabling resources refer to the individual's ability to obtain medical services and the accessibility of medical service resources. Need refers to the direct factors of health service utilisation, including perceived illness measures and evaluated illness measures [19, 20]. The revised Andersen model believes that individual medical behaviour is the result of the interaction of contextual characteristics, individual characteristics and medical results. Since its creation, the Andersen model has been widely used in studies related to health expenditures and health service utilisation and is an authoritative research model for medical and health services [20, 21]. Thus, this study selected the variables based on this model. Furthermore, informal care provided by family members was incorporated into the regression model as an enabling component by referring to the practices of previous studies [22].

## Method and material

### Data and Sample

**Fig. 1:**
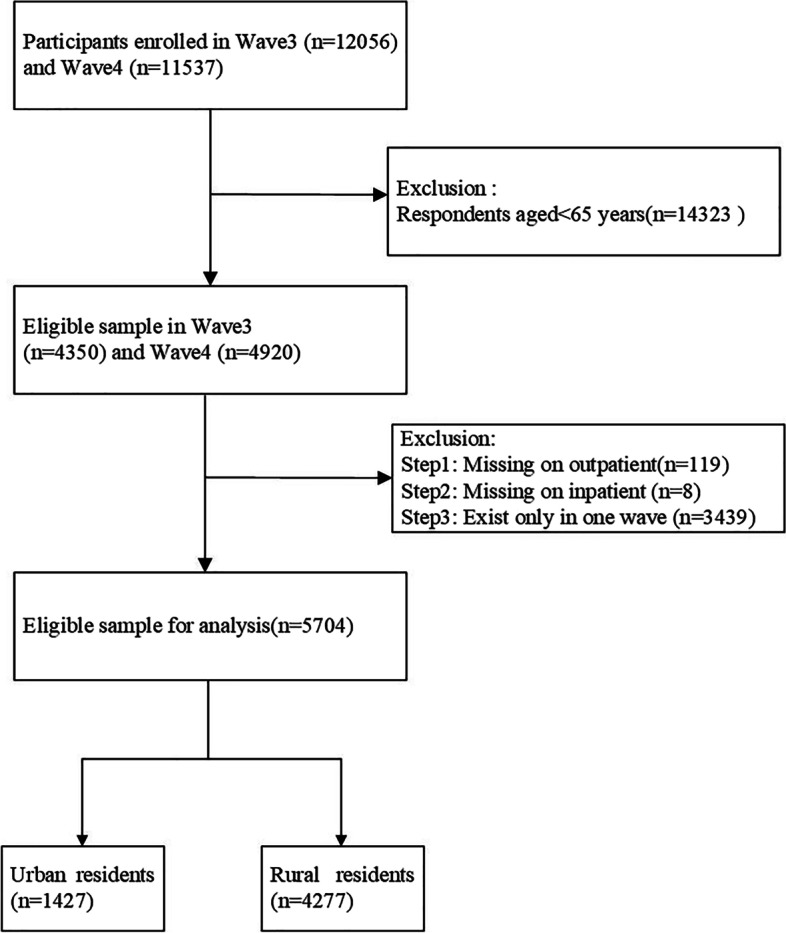
Flowchart of participant selection

The data used in this study were derived from the China Longitudinal Study of Health and Retirement (CHARLS). CHARLS is a national survey aimed at providing comprehensive and high-quality data, such as demographic background, family characteristics, health behaviours and conditions and retirement information of residents aged 45 years and older. A multi-stage stratified proportional probability sampling design was used to select households randomly from 450 villages and residential communities in 150 counties and regions in 28 provinces [23]. The wave 3 study conducted in 2015 and the wave 4 study in 2018 were used for analysis in this paper. A balanced panel data with 5704 participants was formed according to the following criteria: 1) 65 years and older and 2) information about outpatient, hospitalisation and family care were provided in both waves. The details of the sampling process are shown in Fig. 1. The respondents were categorised as urban and rural residents according to the family residential area defined by the National Bureau of Statistics of China.

### Dependent variables

This paper analysed two types of health care utilisation: outpatient and inpatient services. The respondents were asked about how many times they had been visited by medical institutions in the last month and how many times they had received inpatient care during the past year. Outpatient and inpatient visits were used to evaluate the utilisation of health services in the elderly in this study. The dichotomous variables, namely, whether or not the participants use outpatient or inpatient services, were used for the sensitivity test.

### Key variables

Interviewees were asked who helped them with their daily life activities in CHARLS. The 10 options for this question are: a) Spouse; b) Father, Mother, Father-in-law or Mother-in-law; c) Children, Children's spouses, Grandson or Granddaughter; d) Sibling, Brother-in-law, Sister-in-law, Sibling of spouse, Children of sibling, Brother-in-law of spouse, Sister-in-law of spouse, Children of brother-in-law or Children of sister-in-law; e) Other relatives; f) Paid helper (nanny); g) Volunteer; h) Employee(s) of facility; i) Community; j) Others. The participants who selected one or more of options a–e were considered to have accepted informal care from family members [16, 24]. Moreover, the interviewees were asked about the number of days in the past month that family members had assisted them.

In this research, informal care was examined by two aspects:

- Whether the respondents have received informal care from family members. The answers were coded as ‘0 = No’ and ‘1 = Yes.’
- The number of days of informal care that respondents received from all the informal caregivers in a month. According to previous studies, the answers were coded as ‘1 = none,’ ‘2 = less than 15 days,’ ‘3 = between 15 and 29 days’ and ‘4 = 30 days or more’[25].

### The Control variables

Control variables were selected in accordance with a predisposing characteristic, enabling resources and needs according to the Andersen model [26–29]. The following individual-level characteristics were considered as control variables (Table 1).

**Table 1:** Definition/codes of the control variables

	Variables	Codes/definition
Predisposing Characteristics	Gender	0 = Male; 1 = Female
	Age	Continuous variable
	Marital statues	0 = Single; 1 = Partnered
	Education	1 = Illiterate; 2 = Primary school and lower; 3 = Junior middle school; 4 = Senior middle school and higher
Enabling Resources	Medical insurance	0 = None; 1 = Yes
	Pension	Whether received any pension or not: 0 = No; 1 = Yes
	Financial support from children	Financial support received from children: 0 = None; 1 = 0-2000yuan; 2 = 2000-5000yuan; 3 = 5000-10000yuan; 4 = 10000yuan or more
	Smoke	0 = No; 1 = Yes
	Drink	0 = No; 1 = Yes
Need	Chronic diseases	0 = None; 1 = Yes
	Number of ADL limitations	Range from 0 to 6
	Number of IADL limitations	Range from 0 to 6
	Self-rated health	1 = very good; 2 = good; 3 = fair; 4 = poor; 5 = very poor
	Year	2015; 2018

Note: *ADL* activities of daily living; *IADL* instrument activities of daily living

### Statistical analysis

The dependent variables of this study are outpatient visits and inpatient visits, which are count variables; therefore, the analysis methods considered were the Poisson regression and negative binomial regression [30]. However, for a good-fitting model, the Poisson regression requires the mean and variance of the dependent variable to be equal. Substantial departures from this measure may indicate a problem with model specification and also indicate that the estimated standard errors may be downwardly biased. By contrast, negative binomial regression is more suitable when overdispersion occurs [31]. This study examined whether the two dependent variables have overdispersal through the likelihood-ratio test of the alpha value. Results show that the alpha value is significantly not 0 (95% confidence interval [CI]: 4.75–6.03; 95% CI: 1.41–2.09), which meant that the negative binomial regression should be used in this study, instead of the Poisson regression [32, 33]. The negative binomial regression of the panel data includes the fixed effect model, random effect model and mixed effect model. To distinguish which analysis method is suitable for this study, Hausman test is adopted to select between fixed effect model and random effect model, and LR test is adopted to select between random effect model and mixed effect model [34]. Therefor, this study conducted Hausman test (both *P* < 0.001) and LR test (both *P* < 0.001) using inpatient and outpatient visits as the outcome variables, respectively. The results showed that the fixed effect is more applicable. Therefore, fixed-effect negative binomial regression for panel data was conducted to estimate the association between informal care and health service utilisation of the elderly. [35]. Cluster-robust standard errors were used to control heteroscedasticity [36]. The specification of the model was as follows:

$$\mathrm{ln}({\lambda }_{it}) = {\beta }_{1}{\mathrm{care}}_{it}+{\beta }_{2}{P}_{it}+{\beta }_{3}{E}_{it}+{\beta }_{4}{N}_{it}+{\mu }_{it}+\varepsilon ,$$

where *λ*_*it*_ represents the outpatient or inpatient visits of individual *i* in period *t*; care_*it*_ represents the time of informal care obtained by individual *i* in period *t*; and *P*_*it*_, *E*_*it*_, and *N*_*it*_ represent the control variables related to predisposing characteristics, thus enabling resources and needs in the Andersen Model. *μ*_*it*_ is the individual fixed effect, and *ε* is the error term. The incidence rate ratio (IRR) was calculated to facilitate the interpretation of the results. [37].

Model uncertainty is ubiquitous in social science. Thus, substitution-dependent variables and substitution regression models were used to test the robustness [38]. The dichotomous variable, namely, whether outpatient and inpatient services were used, was regarded as the outcome variable. A fixed-effect binary choice model for panel data was used for the sensitivity test.

## Results

### Basic characteristics of the respondents

The basic characteristics of the sample are shown in Table 2. The elderly living in the city received less informal care than those living in the rural areas.

**Table 2:** Sample characteristics of the selected respondents at baseline

	All (*N* = 5704)		Urban(*N* = 1427)		Rural(*N* = 4277)		*P*
	mean (min,max)	Sd	mean (min,max)	Sd	mean (min,max)	Sd	
Inpatient	0.36 (0, 25)	0.93	0.37 (0, 6)	0.81	0.36 (0, 25)	0.97	0.636^c^
Outpatient	0.46 (0, 31)	1.49	0.41 (0, 18)	1.36	0.48 (0, 31)	1.54	0.079^c^
Informal care	1.68 (1, 4)	1.20	1.56 (1, 4)	1.14	1.73 (1, 4)	1.22	< 0.001^a^
Gender	0.49 (0, 1)	0.50	0.51 (0, 1)	0.50	0.49 (0, 1)	0.50	0.108^a^
Age	74.35 (65, 108)	6.54	74.13 (65, 97)	6.36	74.42 (65, 108)	6.59	< 0.001^b^
Marital status	0.49 (0, 1)	0.50	0.53 (0, 1)	0.50	0.48 (0, 1)	0.50	0.004^a^
Education	1.86 (1, 4)	0.87	2.33 (1, 4)	1.08	1.7 (1, 4)	0.72	< 0.001^a^
Chronic diseases	0.46 (0, 1)	0.50	0.47 (0, 1)	0.50	0.45 (0, 1)	0.50	0.340^a^
Smoke	0.26 (0, 1)	0.44	0.2 (0, 1)	0.40	0.27 (0, 1)	0.45	< 0.001^a^
Drink	0.29 (0, 1)	0.46	0.3 (0, 1)	0.46	0.29 (0, 1)	0.45	0.442^a^
Medical insurance	0.92 (0, 1)	0.26	0.94 (0, 1)	0.24	0.92 (0, 1)	0.27	0.042^a^
Number of ADL limitations	0.62 (0, 6)	1.19	0.44 (0, 6)	1.02	0.68 (0, 6)	1.24	< 0.001^c^
Number of IADL limitations	1.23 (0, 6)	1.56	0.74 (0, 6)	1.38	1.12 (0, 6)	1.61	< 0.001^c^
Financial support	1.37 (0, 1)	1.25	1.27 (0, 1)	1.37	1.41 (0, 1)	1.20	< 0.001^a^
Pension	0.16 (0, 1)	0.37	0.47 (0, 1)	0.50	0.06 (0, 1)	0.23	< 0.001^a^
Self-rated health	3.09 (1, 5)	0.94	2.9 ( 1, 5)	0.91	3.15 (1, 5)	0.95	< 0.001^c^

Note: *Sd* standard deviation

^a^outcomes of Chi-square test

^b^outcomes of Student-t test

^c^outcomes of Wilcoxon rank sum test

### Informal care of the elderly in urban and rural areas

**Fig. 2:**
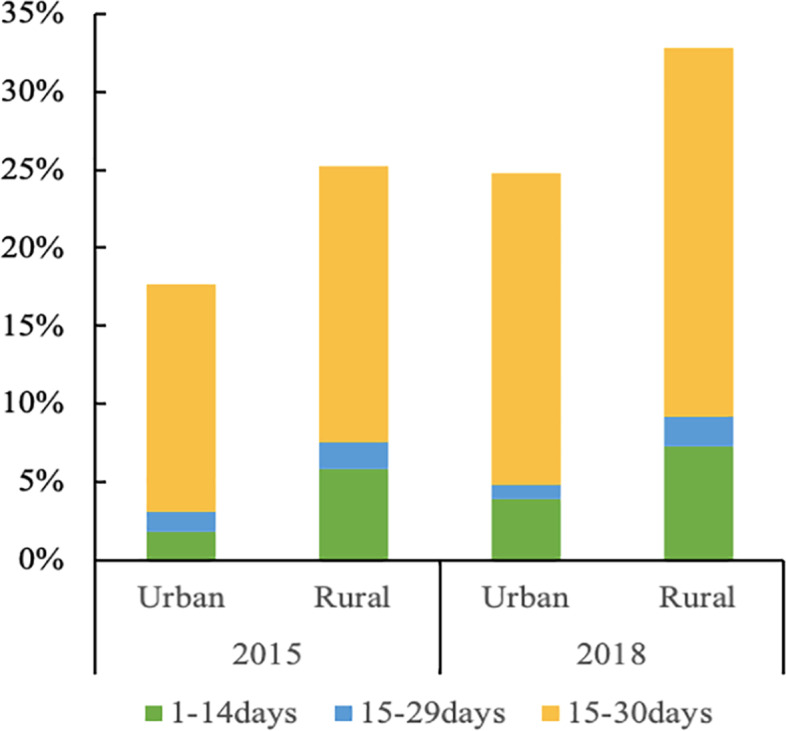
Comparison of informal care in urban and rural areas from 2015 to 2018

This study found by comparing informal care in urban and rural areas of China that the proportion of elderly people who received informal care increased from 19.08% and 28.99% in 2015 to 24.80% and 33.29% in 2018, respectively. The elderly who had been in care for 30 days or more accounted for the highest proportion. Furthermore a higher proportion of rural elderly people received informal care than urban ones in both years (Fig. 2).

### Main results

The results of the negative binomial regression in Table 3 show that informal care provided by family members remarkably improved the utilisation of outpatient and inpatient services for the elderly. Specifically, compared with the elderly who did not receive family care, the inpatient visits of the elderly who received 15–29 days of informal care was higher (IRR = 2.082, *P* < 0.05). Moreover, the elderly who received informal care for more than 30 days had 39.6% more inpatient visits(IRR = 1.396, *P* < 0.01) and 37.4% more outpatient visits than those who did not receive informal care (IRR = 1.374, *P* < 0.05).

**Table 3:** Association between informal care and health service utilisation: fixed-effect negative binomial model for panel data

	Inpatient (*N* = 5704)		Outpatient (*N* = 5704)	
	IRR	Bootstrap standard error	IRR	Bootstrap standard error
Informal care (reference: None)				
1-14d	1.022	0.164	1.219	0.255
15-29d	2.082^b^	0.664	1.262	0.415
30d or more	1.396^c^	0.175	1.374^b^	0.189
Gender (reference: male)	0.583	0.202	1.044	0.263
Age	1.042^b^	0.020	0.944^c^	0.016
Marital status (reference: single)	0.632^b^	0.125	1.290	0.234
Education (reference: illiterate)	1.105	0.113	1.038	0.088
Chronic diseases (reference: None)	1.399^c^	0.109	1.111	0.085
Smoke (reference: No)	0.470^c^	0.081	0.869	0.158
Drink (reference: No)	0.720^b^	0.116	1.028	0.132
Medical insurance (reference: None)	1.275	0.291	1.263	0.215
Number of ADL limitations	1.056	0.041	1.037	0.042
Number of IADL limitations	1.044	0.042	0.993	0.039
Financial support (reference: None)	1.107^b^	0.044	0.939	0.039
Pension (reference: None)	0.911	0.147	1.331^a^	0.228
Self-rated health	1.238^c^	0.071	1.267^c^	0.070
Intercept	0.026^c^	0.036	7.624	10.204

Note: *IRR* incidence rate ratio

(^a^), (^b^), (^c^) mean significant level at 10%, 5%, 1%, respectively

*ADL* activities of daily living, *IADL* instrument activities of daily living

The samples were divided into urban and rural residents to test the difference in the association between informal care and formal care of the elderly in urban and rural areas. The results in Table 4 show the remarkable differences between urban and rural areas. In terms of inpatient visits, the number of hospitalisations of the elderly in rural areas who received care for 15–29 days and more than 30 days increased by 111.4% (IRR = 2.114, *P* < 0.05) and 53.2% (IRR = 1.532, *P* < 0.01), respectively. In terms of outpatient visits, urban elders who received informal care within 15 days had increased outpatient visits (IRR = 3.164, *P* < 0.01). For rural elderly who received informal care for 30 days or more, informal care was positively associated with outpatient visits (IRR = 1.500, *P* < 0.05).

**Table 4:** Association between informal care and health service utilisation in urban and rural China: fixed-effect negative binomial model for panel data

	Urban (*N* = 1427)		Rural (*N* = 4277)	
	IRR (Inpatient)	IRR (Outpatient)	IRR (Inpatient)	IRR (Outpatient)
Informal care(reference: None)				
1-14d	0.488	3.164^a^	1.210	1.330
15-29d	2.038	2.015	2.114^b^	1.227
30d or more	1.208	1.804	1.532^c^	1.500^b^

Note: *IRR* incidence rate ratio. All models have controlled variables, including gender, age, marital status, education, chronic diseases, smoke, drink, medical insurance, number of ADL limitations, number of IADL limitations, financial support, pension and self-rated health. (^a^), (^b^), (^c^) mean significant level at 10%, 5%, 1%, respectively

### Sensitivity tests

This study regarded whether the respondents used outpatient and inpatient services or not as outcome variables to test the robustness of the results. A fixed-effect binary choice model for panel data was selected after the Hausman test (both *P* < 0.001). The result in Table 5 shows that informal care can remarkably increase the utilisation of outpatient and inpatient services for the elderly in rural areas. The result is consistent with the outcome of the negative binomial regression model.

**Table 5:** Association between informal care and health care utilisation in urban and rural China: fixed-effect binary choice model for panel data

	Urban (*N* = 1427)		Rural (*N* = 4277)	
	Inpatient	Outpatient	Inpatient	Outpatient
Informal care(reference: None)				
1-14d	-1.375	0.96^a^	0.248	0.404
15-29d	1.022	-0.061	1.058^b^	0.174
30d or more	0.9	0.001	0.561^b^	0.481^b^

Note: All models have controlled variables, including gender, age, marital status, education, chronic diseases, smoke, drink, medical insurance, number of ADL limitations, number of IADL limitations, financial support, pension and self-rated health. (^a^), (^b^), (^c^) mean significant level at 10%, 5%, 1%, respectively

## Discussion

### Status of informal care and health care utilisation

The proportion of informal care in China is increasing, and the proportion of informal care in rural areas is higher than that in urban areas. The reason may be that Chinese families follow The Pecking Order Theory in their choice of care mode for the elderly, which meant that they will use all internal care resources first before using formal in- or outpatient health care [39].

The elderly living in the city have slightly more outpatient visits and lesser inpatient visits than those of the rural elderly. This result may be due to the fact that the urban elderly have more convenience to seek medical treatment and more channels to receive outpatient services than the rural elderly [40]. The rural elderly’s priority health needs are inhibited by personal economic conditions, especially for diseases with mild symptoms, which may reduce their outpatient visits [41]. Furthermore, mild symptoms may become severe because of the poor economic conditions and poor health literacy of rural elderlies. Therefore, the hospitalisation rate is high in rural areas [42].

### Association between informal care and formal care

This study found that informal care provided by family members is positively associated with the utilisation of outpatient and inpatient services for the elderly. Similar findings were found in previous studies [15, 16, 43]. Accepting informal care for 15 days or more can remarkably increase the utilisation of health services for the elderly.

In terms of inpatient care, the elderly who receive informal care for 15–29 days per month have more than twice as many inpatient services as those who do not receive care. The hospitalisation rate of the elderly who received informal care for 30 days or more is higher by 39.6% compared with those who did not receive care. On the one hand, this may be due to the fact that hospitalisation involves highly professional and highly skilled personnel, who cannot be replaced by informal care [15]; on the other hand, informal caregivers may detect the diseases of the care recipients in time and help them seek medical attention promptly.

In terms of outpatient care, the elderly who receive informal care for 30 days or more have a higher frequency of outpatient use. The reason may be that informal care provided by family members is more related to daily life care than medical services. Accordingly, caregivers can consult doctors about health status and medical treatment of the elderly to reduce medical barriers. Moreover, the elderly can be sent to medical institutions in time because of the preventive psychology of informal caregivers [44].

Results indicated that informal care can release the elderly's demand for medical services and enhance the accessibility of medical services. In addition to making relevant policies from the perspective of economic input to fulfil the medical service needs of the elderly, relevant policies should also be made from the perspective of caregiving resources.

### Heterogeneity between urban and rural areas

The effect of informal care on health care utilisation differs greatly between urban and rural areas in China. This result is consistent with previous studies [45, 46]. The elderly living in cities receive informal care without affecting their inpatient visits. However, in rural areas, receiving care from family members can increase the hospitalisation rate of the elderly. In terms of outpatient use, informal care within 15 days can increase the number of outpatient visits of the urban elderly, while informal care of 30 days or more can increase the outpatient visits of the rural elderly. This difference may be due to the dual socioeconomic structure of the urban–rural divide and rule; urban and rural elderly people have differences in income, concepts and service provision [47]. The elderly in rural areas have poor medical awareness and poor economic conditions; therefore, family members have a greater role in urging the elderly to seek medical treatment [40]. Another reason may be the better accessibility of health resources for the urban elderly than that of the rural elderly [10]. The elderly in rural areas, especially the disabled, require help from other family members to obtain outpatient and inpatient services because of the long distance to health care facilities.

Informal care plays an important role in the utilization of medical services for the elderly. In China, especially in rural areas, caring for the elderly is the responsibility and moral obligation of other family members. The culture of filial piety has a deep effect on the family. However, the income of rural families in China is generally lower than that of urban families. In this case, providing informal care for the elderly has brought plenty of economic pressure to caregivers. Therefore, while encouraging informal care, policymakers should also pay attention to the differences between urban and rural care systems. Relevant policies should protect the interests of informal caregivers, especially in rural areas.

### Limitation

Three limitations exist in this study. Firstly, the study period covers only two waves. The short study period may not reflect the long-term effects of informal care on health care utilisation. Secondly, the endogeneity in this study was not controlled which may lead to biased estimates. Lastly, this study focuses on informal care provided by family caregivers instead of different caregivers.

## Conclusion

This study has two main findings. Firstly, informal care provided by family members can remarkably increase the outpatient and inpatient visits of the elderly. Secondly, the association between informal and health care utilisation varies between rural and urban residents. The association between informal care and the health care use amongst urban elderly is weaker than that in rural areas. These findings indicated the role of informal care and reminded that relevant departments should pay attention to the value of informal care and consider the differences between urban and rural elderly groups. Policymakers should formulate different care policies according to the needs of health services for the elderly in urban and rural areas, and promote rational use of health services for the elderly, especially in rural areas.

## Data Availability

The datasets generated and analysed during the current study are available in the CHARLS repository, [http://charls.pku.edu.cn/en].

## Abbreviations

CHARLS: China Longitudinal Study of Health and Retirement
IRR: Incidence rate ratio
Sd: Standard deviation
ADL: Activities of daily living
IADL: Instrument activities of daily living
SE: Standard error
